# Facilitating healthcare decisions by assessing the certainty in the evidence from preclinical animal studies

**DOI:** 10.1371/journal.pone.0187271

**Published:** 2018-01-11

**Authors:** Carlijn R. Hooijmans, Rob B. M. de Vries, Merel Ritskes-Hoitinga, Maroeska M. Rovers, Mariska M. Leeflang, Joanna IntHout, Kimberley E. Wever, Lotty Hooft, Hans de Beer, Ton Kuijpers, Malcolm R. Macleod, Emily S. Sena, Gerben ter Riet, Rebecca L. Morgan, Kristina A. Thayer, Andrew A. Rooney, Gordon H. Guyatt, Holger J. Schünemann, Miranda W. Langendam, on behalf of the GRADE Working Group

**Affiliations:** 1 Systematic Review Centre for Laboratory Animal Experimentation (SYRCLE), Department of Health Evidence, Radboud University Medical Center, Nijmegen, The Netherlands; 2 Department of Clinical Epidemiology, Biostatistics and Bioinformatics, Academic Medical Center, University of Amsterdam, Amsterdam, The Netherlands; 3 Cochrane Netherlands, University Medical Center, Utrecht, The Netherlands; 4 Guide2Guidance, Urecht, The Netherlands; 5 Dutch College of General Practitioners, Utrecht, The Netherlands; 6 Center for Clinical Brain Sciences, University of Edinburgh, Edinburgh, United Kingdom; 7 Department of General Practice, Academic Medical Center, University of Amsterdam, Amsterdam, The Netherlands; 8 Department of Health Research Methods, Evidence, and Impact, McMaster University, Hamilton, ON, Canada; 9 Department of Medicine, McMaster University, Hamilton, Canada; 10 Division of the National Toxicology Program, National Institute of Environmental Health Sciences, National Institutes of Health, Department of Health and Human Services, Washington, D.C., United States of America; University of Münster, GERMANY

## Abstract

Laboratory animal studies are used in a wide range of human health related research areas, such as basic biomedical research, drug research, experimental surgery and environmental health. The results of these studies can be used to inform decisions regarding clinical research in humans, for example the decision to proceed to clinical trials. If the research question relates to potential harms with no expectation of benefit (e.g., toxicology), studies in experimental animals may provide the only relevant or controlled data and directly inform clinical management decisions.

Systematic reviews and meta-analyses are important tools to provide robust and informative evidence summaries of these animal studies. Rating how certain we are about the evidence could provide important information about the translational probability of findings in experimental animal studies to clinical practice and probably improve it. Evidence summaries and certainty in the evidence ratings could also be used (1) to support selection of interventions with best therapeutic potential to be tested in clinical trials, (2) to justify a regulatory decision limiting human exposure (to drug or toxin), or to (3) support decisions on the utility of further animal experiments. The Grading of Recommendations, Assessment, Development, and Evaluation (GRADE) approach is the most widely used framework to rate the certainty in the evidence and strength of health care recommendations. Here we present how the GRADE approach could be used to rate the certainty in the evidence of preclinical animal studies in the context of therapeutic interventions. We also discuss the methodological challenges that we identified, and for which further work is needed. Examples are defining the importance of consistency within and across animal species and using GRADE’s indirectness domain as a tool to predict translation from animal models to humans.

## Introduction

### Systematic reviews of animal studies

Laboratory animal studies are used in a wide range of human health related research areas, such as basic biomedical research, drug research, experimental surgery and environmental health. The results of these studies can be used to inform human research, for example to unravel pathophysiology and mechanisms of action of treatment and to select therapeutic interventions to be tested in clinical trials. If the research question relates to potential harms with no expectation of benefit (e.g., toxicology), studies in experimental animals may provide the only relevant data.

However, systematic reviews (SRs) can provide robust and informative summaries of animal studies [1]. In a rigorous SR reproducible identification, selection, appraisal and analysis are used to summarize the relevant evidence in order to support well-informed decisions in healthcare [2].

SRs of animal studies are relatively novel. Published guidance is emerging on how to develop a protocol for a SR of animal studies, comprehensively search for experimental animal studies, appraise the risk of bias of the included studies, and perform meta-analysis (MA) to estimate the pooled effect of the interventions [3–11]. Table 1 presents a comparison of the differences on of current status of systematic reviews (with focus on meta-analysis) of human and animal research. The first international symposium on SRs in laboratory animal science was organised by Systematic Review Center for Laboratory Animal Experimentation (SYRCLE) in 2012, and a journal dedicated to publishing SRs of animal studies, Evidence Based Preclinical Medicine [http://onlinelibrary.wiley.com/journal/10.1002/(ISSN)2054-703X/homepage/EditorialBoard.html], was launched in 2014.

**Table 1:** Comparison of current status of systematic reviews (with focus on meta-analysis) of human and animal research.

	SR of human studies (RCTs)	SR of animal studies
General goal of the meta-analysis	Estimate the overall effect size of a consistently applied intervention to aid decision making in clinical practice and to assess if effects are consistent across similar or different populations and settings.	To explore heterogeneity to generate new hypotheses about pathophysiology and treatment, to guide (some aspects of) the design of new clinical trials and to test efficacy and safety of an intervention.
Summarizing effects across studies	Often pooled effect (direction and size) because of more precise selection of the PICO elements within the research question. (PICO: Patients/people, Intervention(s), Comparator(s) and patient important Outcomes).	Generally only direction of pooled effect (based on confidence interval). Because of unavoidable heterogeneity the point estimate is difficult to interpret.
Summary effect measure for continuous data	Mean difference is preferred because of the ease of interpretation over standardized mean difference (SMD). SMD is used if outcomes are measured using different outcome measures or approaches.	Normalized mean difference (NMD [8]) and SMD are usually provided because of large variation in outcomes, outcome assessment and differences in outcomes between species.
Options for exploring heterogeneity	Intentionally limited to enhance certainty in the effect estimates.	Wider range of options to examine toxicity, pathology, and mechanisms of disease, and therefore greater potential for exploring possible sources of heterogeneity.
Amount of statistical heterogeneity	Varies between meta-analyses	Substantial in almost all meta-analyses
Reporting standards and risk of bias assessment within primary studies	Established guidelines. Quality of reporting of recent RCTs is relatively high. Risk of bias varies.	Recently introduced guidelines. Quality of reporting often poor. Risk of bias seems considerable (e.g. lack of blinding, inadequate randomization) but is often difficult to assess because of inadequate reporting.

SRs of animal studies can facilitate healthcare decisions such as selection of interventions with therapeutic potential to be tested in clinical trials, regulatory decisions limiting human exposure (drugs or toxicants) or decisions on the utility of further animal studies. In addition, evidence from animal studies can inform clinical management decisions, if other evidence is lacking. The level of certainty in the evidence plays an essential role in these decisions, but guidance regarding the assessment is lacking.

An important next step is, therefore, to develop guidance on how to interpret the results of a SR of animal studies and to rate the certainty in the evidence (also called the quality of evidence or the confidence in an effect estimate). Meta-analysis of the included studies and assessment of the certainty in the evidence might contribute to improving the probability of translating findings in experimental animal studies to human studies and clinical practice. Following this hypothesis, a higher certainty in the evidence would be related to higher success rates in applying the results to human clinical questions (i.e. higher translatability).

### GRADE

The Grading of Recommendations, Assessment, Development, and Evaluation (GRADE) approach is the most widely used method to rate the certainty in the evidence and strength of health care recommendations [12–15]. GRADE starts with a focused question and specification of patient relevant outcomes [16]. For each outcome, the certainty in the evidence is assessed across studies and can be rated as high, moderate, low or very low. For interventions, evidence from randomized controlled trials (RCTs) starts as high quality evidence. Evidence from non-randomized (observational) studies is downgraded to low quality evidence because of lack of appropriate protection against uncontrollable bias and confounding. Then, within the study categories, additional domains that may reduce our certainty in the evidence are considered: risk of bias, unexplained inconsistency of results (indicated by statistical heterogeneity), imprecise results (e.g. wide confidence intervals, small event rate and sample size), indirect results (indirectness) and publication bias. Similarly, for methodologically rigorous non-randomized (observational) studies, large effects, a dose-response relation and the expected effect of plausible residual confounding may lead to upgrading of the initial certainty level [17–23].

For systematic reviews, the endpoint of a GRADE assessment is a rating of the certainty in the evidence for each outcome. A summary of evidence, which includes this rating, is presented in GRADE evidence profiles (EP) or Summary of Findings (SoF) tables [24–27].

To arrive at an evidence-based healthcare decision, information additional to the evidence of the effectiveness is needed. GRADE therefore offers ‘Evidence to Decision’ (EtD) frameworks [28–31]. Grading of recommendations–for or against and strong or conditional–is based on the overall certainty in the evidence, balance between desirable and undesirable effects, patient or population values and preferences, resources, acceptability, equity, and feasibility [32, 33].

### Aim of the paper

The aim of this paper is to explore how the GRADE framework can be used to assess the certainty in the evidence of preclinical animal intervention studies. Preclinical animal intervention studies are experimental studies in which the investigator controls the intervention [34]. These studies are typically used to test the efficacy and safety of medical interventions, for example in the preclinical phase of the development of new drugs, or to better understand disease or intervention mechanisms or the action of the intervention. The paper focusses on assessment of the evidence. How to move from evidence to decision, i.e. taking into account other factors than the evidence, will be addressed in a separate paper.

Although this paper is about evaluating the body of evidence of preclinical animal studies in the context of clinical treatment, the presented GRADE approach could possibly be applied to evidence from animal studies in the field of toxicology and environmental health, but further research is needed [35].

Intended users of the framework are systematic reviewers and others interested in transparent and explicit evaluation of experimental animal evidence, for example preclinical and clinical researchers, pharmaceutical companies, potential funders of clinical or animal research and ethical committees.

## Methods

The proposed GRADE approach to assess the certainty in the evidence from preclinical animal intervention studies is based on the main principles of GRADE[25] and was developed in a 6-step process. Step A and B focused on identifying potential additional domains and additional aspects within the existing domains that may reduce our certainty in the evidence.

Step A: We surveyed how authors of SRs of animal studies addressed certainty in the evidence. The starting point was a recently published SR on all instruments that assess risk of bias and other methodological criteria in animal research [36]. This review identified 30 instruments with a total of 219 criteria of which 66 seemed to be unique. Two authors (MWL and CH) selected criteria on their possible relevance for judging certainty in the evidence of animal studies (e.g. only excluding clearly irrelevant criteria concerning ethical issues, reporting quality or statements made about whether or not 1) housing details of the animals are described or 2) a methods section was included in the manuscript) and retained 32 criteria (S1 Table).

Step B: To identify additional GRADE (sub)domains, we surveyed which aspects of certainty in the evidence are currently assessed and reported in SRs of animal intervention studies. We therefore performed a systematic literature search (see Box 1 for the search strategy and eligibility criteria). The aim of the literature search was to find evidence for potential additional GRADE domains (or aspects of domains). For this reason, we restricted the search to more recently published SRs because we assumed these were of higher (reporting) quality compared to earlier published SR. We assessed a random sample and not the full set of SRs that fulfilled the inclusion criteria because we expected to reach saturation in finding potential additional domains and aspects in a subset of all SRs.

**Box 1 Search strategy and eligibility criteria**

**Search**

Two component Systematic PubMed Search:

Component 1: Systematic reviews: systematic review[ti] OR systematic reviews[ti] OR meta-analysis[pt] OR meta-analyses[ti] OR meta-analysis[ti] OR systematic literature review[ti] OR (systematic review[tiab] AND review[pt]) OR Systematic survey[tiab] OR Critical survey[tiab] OR Systematic surveys[tiab] OR (systematic overview[tiab] OR systematic overviews[tiab]) OR critical overview[tiab] OR systematic review[tiab] OR systematically review[tiab] OR critical review[tiab] OR systematic reviews[tiab] OR critical reviews[tiab]) NOT (letter[pt] OR newspaper article[pt] OR comment[pt]).

Component 2: animal studies: Pubmed searchfilter (ref)

Filters used: Publication date from 2012/01/01 to 2013/12/01.

**Inclusion criteria**: systematic review or meta-analysis in title; aim of SR was to investigate an intervention with aim to improve human medicine; pdf available in Radboud Medical Library

**Selection procedure**:

- Selection of papers was alphabetically ordered and each 5th paper was initially selected to be screened for details of the quality assessment;
- In case the selected paper did not assess study quality in the method section, also the chronologically following paper was selected and screened.

The search resulted in 204 SRs of preclinical animal intervention studies, and random sample of 88 SRs were examined. Three authors (MWL, KEW and CRH) studied the Methods sections of these papers and identified 1) whether a methodological quality assessment had been conducted (using risk of bias and/or other methodological criteria) and which methodological criteria had been taken into account, and 2) if (and which) methodological quality indicators were used as eligibility criteria. Thirty-nine possibly relevant criteria (S2 Table) were identified. In 31 of 88 (35%) systematic reviews, methodological quality assessment had been performed. From these 31 SRs, CRH, MWL and KEW screened the Discussion sections and identified 37 criteria (S2 Table) possibly relevant for the interpretation of the results and reaching a conclusion related to the health care question.

In summary, the analysis of both the tools presented in the paper of Krauth et al, and the methods and discussion section of random selected systematic reviews of animal studies resulted in 108 criteria (n = 32 +n = 39 + n = 37). After removing the duplicate criteria, 59 criteria remained. Most of these criteria (n = 44) could be classified in one of the eight GRADE domains (risk of bias, unexplained inconsistency of results, imprecise results, indirect results, publication bias, dose response relationship, large effects, confounding likely to minimize effect. The criteria that could not be classified in a GRADE domain were not related to certainty in the evidence or duplicates (S3 Table).

Step C: To identify challenges in applying the ‘human studies’ GRADE approach to animal research four authors (RBMdV, KEW, HB, MWL and CRH) applied GRADE to a selection of three SRs of preclinical animal intervention studies on different topics.

Step D: Based on the previous steps and the GRADE approach for human studies MWL and CRH developed the draft GRADE approach for preclinical animal intervention studies.

Step E: In several brainstorm sessions, two group expert meetings and two consultations with experts (see S1 File), application of GRADE to evidence from animal studies was discussed, the draft approach was improved, and challenges and research needs were identified. The expert group consisted of systematic review experts (for clinical and animal studies), GRADE methodologists and a statistician.

Step F: To illustrate the practical use of the approach, and some of its challenges, we applied the drafted GRADE approach to an existing systematic review of animal studies on the use of probiotics for severe acute pancreatitis [37].

## Results

Steps A to F resulted in the GRADE approach for assessing the quality of the evidence from preclinical animal intervention studies in the context of clinical treatment.

### Applying GRADE to preclinical animal evidence

The proposed GRADE approach for preclinical animal evidence (Box 2) follows the main principles of GRADE [25]. However, in animal studies, the following issues need special consideration: 1) how well do the results translate from animals to the clinical situation (in GRADE terminology called indirectness), 2) operationalizing of within and between-species inconsistency, 3) upgrading (when to upgrade or how to apply the factors), and 4) the content of the GRADE EP.

**Box 2 Steps in the GRADE approach for preclinical animal intervention studies**

1. Define and frame the clinical question (patients/people, intervention(s), comparator(s) and patient important outcomes (clinical PICO))
2. Scope the evidence and proceed with next steps if evidence from animal studies is needed to answer the question
3. Formulate preclinical PICO (specify animal model(s), intervention, comparator and outcomes related to the patient important outcomes defined in the clinical PICO)
4. Collect animal studies evidence and summarize the effect estimates by outcome
5. Assess and rate the quality of the evidence from animal studies by outcome and relate these to the clinical question
  - Assess risk of bias, imprecision, inconsistency and publication bias
  - Assess indirectness for
    ■ Evidence from preclinical animal studies compared to preclinical PICO
    ■ Evidence from preclinical animal studies compared to clinical PICO (also called translatability)
  - Assess factors that may lead to upgrading
  - Rate the certainty in the evidence for each outcome considering all GRADE domains

### Step 1: Framing and rationale of the clinical question

In the proposed approach (Box 2), the overall goal is to answer a clinical question. The first step is to specify the health care question using the PICO methodology. Clinical (therapeutic) PICOs address the comparison of an intervention with a relevant comparator (e.g. no treatment/placebo or another intervention). The outcomes are specified according to their importance to patients/population, and should include benefits and harms (or desirable and undesirable health effects) of the intervention [16].

For the probiotics for pancreatitis example, the clinical PICO was: “What is the impact of probiotic prophylaxis (I) compared to no probiotic prophylaxis (C) in patients with predicted severe acute pancreatitis (P) on infectious complications, mortality, (multi)organ failure, need for surgical intervention, antibiotic resistance, increased hospital stay, abdominal complaints and adverse events) (O)”.

### Step 2: Scoping the evidence

The second step (Box 2) is to scope the literature to assess the pertinent clinical evidence. When there is no or very limited (quality) evidence from human studies, the GRADE approach can be used to answer the question using evidence from animal studies. Reasons for synthesizing animal evidence include the intervention still being in development (never tested in humans, preclinical phase), or that clinical experiments are unethical, for example because of the nature of the intervention or nature of the outcomes and in the absence of observational studies in humans. In case of very low quality human evidence, considering evidence from animal studies might change the assessment of likely magnitude of effect or might potentially increase our certainty in the evidence.

For the probiotics for pancreatitis example, we assume that the intervention has not been tested in humans.

### Step 3: Formulate the preclinical PICO

The third step is to formulate the preclinical PICO (Box 2). As the clinical question is leading, the preclinical PICO should be derived from the clinical PICO. A separate preclinical PICO is needed to define the eligibility criteria for the SR of animal studies. It also serves as a first outline of aspects of indirectness. To reduce indirectness, it is important to closely collaborate with clinical experts and to describe how the preclinical outcomes are linked to the patient-important outcomes (see step 1). Table 2 presents both PICOs.

**Table 2:** Clinical and preclinical PICO elements.

Clinical PICO (Question of interest)	Preclinical PICO (Question in preclinical context)
Patient/people	Animal model(s) (species) and method of induction of disease (if relevant) should represent the patient population
Intervention(s)	Intervention should reflect clinical practice as much as possible
Comparator(s): No treatment or another intervention	Control group: No treatment, vehicle/placebo or sham treatment, or another intervention
Ideally outcomes directly important to people. In practice surrogate outcomes (e.g. lab values) are used as well.	The outcomes should be relevant to the clinical situation. In preclinical animal intervention studies not all outcomes have to be directly relevant to patients, depending on what level of indirectness one wants to accept. Surrogate outcomes can be relevant if they measure a biological effect or mechanism that is difficult to assess precisely in patients for ethical reasons (invasive and/or potentially harmful).

In the probiotics for pancreatitis example, the preclinical PICO was defined as:

P: laboratory animals with induced acute pancreatitis (all species)

I: probiotic treatment

C: no probiotics or vehicle only

O: mortality, histopathology of the pancreas, bacterial translocation to the pancreas and mesenteric lymph nodes.

One of the important clinical outcomes in severe acute pancreatitis is the number of infectious complications. This is because secondary infection as a consequence of an inflamed pancreas is considered to be the main cause of death. In animal experiments, histopathology of the pancreas and bacterial translocation are the most representative outcomes because the animals are sacrificed at the end of the experiment and entire organs can be extracted and studied to provide more detailed information about the nature and presence of infectious complications [37].

### Step 4: Collect preclinical evidence and summarize effect estimates by outcome

The optimal application of GRADE requires an up-to-date and well-conducted SR of animal studies [18, 25]. A search for existing SRs can be performed, or a new SR can be conducted [6, 8]. The eligibility criteria follow from the preclinical PICO.

A GRADE EP summarizes the SR results. Standard elements are the number of studies and participants, study design, relative effect, baseline risk (risk in the control group) and corresponding absolute effect and certainty in the evidence [38, 39].

We identified several issues in developing an EP to summarize the results of animal SRs. Some of these issues reflect differences in the current status of developing SRs in human and animal research. Examples of these differences (with focus on MA) are presented in Table 1.

To calculate absolute effects, defining a baseline risk is needed. When the aim of the review is to inform clinical decisions the clinical baseline risk might be most appropriate. If the aim of the review is to explore if an intervention could be promising for clinical use a baseline risk based on animal data, which relates directly to the estimated effect, might facilitate interpreting the results of the animal studies.

Another issue is presentation of the summarized treatment effect (such as pooled relative effect, mean difference or standardized mean difference) in the light of the often substantial amount of variability and statistical heterogeneity among studies in SRs of animal studies.

In SRs of animal studies, variability in species, interventions and/or outcomes is often deliberately chosen as the aim of these reviews are explorative. Including different species is common because there is currently no evidence-based approach of how to choose the preferred animal model(s) that would answer the clinical question best.

Reasons for heterogeneity are explored using subgroup analyses to provide important information to generate new hypotheses and guide the design of clinical trials. No statistical heterogeneity may indicate that the effects are broadly generalizable, or can simply mirror that the intervention has only been tested under the most propitious circumstances, and that further research is required. In human SRs the ideal situation is no heterogeneity. In animal SRs what can be considered as acceptable amount of heterogeneity is not known, but merits further investigation.

Interpretation of the pooled effect size as the estimated treatment effect may not be appropriate in case of substantial statistical heterogeneity. One could argue that proceeding to clinical trials is only sensible when the pooled effect in animal studies is sufficiently large and meaningful for the clinical situation. In that case, the magnitude of the effect and surrounding heterogeneity are relevant. Depending on the amount of statistical heterogeneity, authors may consider presenting the pooled effect size, effect size categories (small, moderate, large) or the direction of the effect in the GRADE EP.

To avoid inappropriately pooled effects, meta-analysts need to carefully explore inconsistency and avoid statistical synthesis of too heterogeneous data. In animal research this would mean, for example, choosing the animal model that would answer the clinical question best, or to restrict the variations in the intervention or outcomes. However, as explained above, this is influenced by the aim of the review.

In the probiotics for pancreatitis example, the mortality risk in the animal studies was lower in the probiotics group, but the confidence interval (CI) was very wide and included no effect (3 studies, 102 rats, OR 0.54, 95% CI 0.24–1.22). The risk in the control animals undergoing no treatment or placebo treatment (baseline risk based on animal data) was 47.2%. A baseline risk for mortality in patients could be estimated at 10–30% [40].

### Step 5: Assess and rate the quality of a body of the animal research evidence by outcome

#### Initial study design

Preclinical animal intervention studies are experimental studies in which the investigator controls the intervention [34]. In our approach (Box 1) evidence from randomized animal experiments start as high quality evidence. Inadequate or lack of randomization is part of the risk of bias assessment. The identification of, and classification of different types of study design in preclinical animal research merits further research. For example, cross-over designs, in which all animals receive all treatments, or non-experimental (i.e. observational) animal studies to assess ecological impacts of an exposure occur in the environmental health setting. It is unclear, however, for which types of questions these study designs are used in preclinical animal studies.

In rating the certainty of the evidence we propose to assess–by outcome–the GRADE downgrading factors a) risk of bias, imprecision, inconsistency and publication bias, followed by b) two layers of indirectness and c) considering upgrading (Box 1). The last step is to rate the certainty in the effect taking all factors in conjunction. How indirectness should be weighted in the total rating remains a challenge.

#### Step 5a: Assess risk of bias, inconsistency, imprecision and publication bias

Trials may incur risk of misleading results if they are flawed in their design or conduct. Therefore, the risk of bias of the individual studies needs to be assessed as one of the steps of performing a SR. There are several tools available to assess risk of bias of animal studies [36], for example the SYRCLE risk of bias tool [4]. S4 Table shows the main differences between risk of bias of clinical and animal intervention studies. Second, for each outcome the risk of bias across all included studies (body of evidence) is assessed.

Although rigorous SRs demand assessment of risk of bias of the included studies, currently this is not common practice in SR of animal studies [41]. This implies that assessment of risk of bias may still need to be performed if one uses existing SRs. Poor reporting of essential design characteristics of the animal experiments may often hamper this step.

In the probiotics for pancreatitis example, risk of bias was assessed with SYRCLE’s risk of bias tool [4]. Risk of bias was unclear in the majority of the studies due to poor reporting. To tackle this may require contacting study authors.

Inconsistency is typically assessed by considering the overlap between confidence intervals (CI), the magnitude and direction of effect of the individual studies, the p-value of the test for heterogeneity and I^2^ (describes the percentage of the variability in effect estimates that is due to heterogeneity rather than sampling error (chance)) [21]. The GRADE approach suggests rating down the certainty in the evidence if inconsistency in the results of the individual studies remains unexplained after exploration of hypotheses that might explain heterogeneity. If differences in population, intervention, comparators or outcomes or risk of bias provide an explanation for heterogeneity, meta-analysts should offer estimates for the appropriate subgroups (or, if risk of bias explains the inconsistency, use of only the low risk of bias studies might be appropriate).

Several challenges remain in assessing inconsistency. First, heterogeneity in animal research can be expected, as a result from the often exploratory approach. In other words, part of the heterogeneity is intentionally induced and, in that case, should not be part of the certainty of the evidence grading because it can be explained. The issues with regard to inconsistency are: a) how to separate induced and explained from unexplained heterogeneity; and b) how to interpret I^2^. Second, heterogeneous results overall could be consistent within species, therefore two levels of inconsistency can be present: within species and across species. For example, when all species included in the analysis show the same direction of effect, we are more certain that the intervention effect is robust across different species, including humans. In that case we would not downgrade for inconsistency even if the results overall are heterogeneous.

In the probiotics for pancreatitis example, inconsistency was not a concern for mortality: the CIs of the three studies were overlapping and there was no statistical heterogeneity as indicated by an I^2^ of 0.0% and a p value of 0.73. The three studies were conducted in rats. For the histopathology outcomes (6 studies in rats) the CIs did not overlap and I^2^ was 85% (p-value <0.01), indicating substantial heterogeneity. The results of five out of the six studies favored probiotics. However, the effect size (odds ratios) varied greatly among those five studies. For histopathology, inconsistency is a concern and was a reason for downgrading.

To judge imprecision, GRADE suggests focusing on the 95% CIs around the effect estimate and calculating the optimal information size (OIS, the number of patients required for an adequately powered individual trial). Results are imprecise when based on relatively few patients/animals and few events, which usually leads to wide CIs around the (summary) estimate of the effect. For SRs, results are precise if the OIS is met and the CI excludes no effect, or if the CI overlaps with no effect and the CI is very narrow (precise estimate of no effect). GRADE suggests rating down if the OIS is not met, or if the OIS is met and the CI overlaps no effect and fails to exclude important benefit or important harm. This implies setting thresholds for clinically relevant differences [17].

The most important issues for animal studies are how to calculate the OIS and set meaningful thresholds for clinical relevance. In animal intervention studies the experimental unit can be the cage, as for ethical welfare reasons individual housing of the animals is not always possible [34]. This resembles cluster randomization in human research, for which power calculation methods are available. Taking the experimental unit into account in calculating the OIS in animal studies needs further exploration.

Furthermore, as explained in step 4, in interpreting the results of preclinical animal studies, the direction of the effect is often perceived to be more important than the exact magnitude of effect. In that case judgment of imprecision will be based on whether the confidence interval includes no effect or not.

If effect size matters, one could consider categories of effect size (e.g. SMD effect sizes below 0.2 are small, between 0.2 and 0.5 moderate and above 0.8 large). While absolute rules are not available, raters could consider downgrading for imprecision if the CI overlaps two or more categories; in such instances an appropriate explanation should be provided. Another suggestion could be to set the thresholds based on the effect sizes of effective drugs, if such information is available.

As for GRADE applied to human research setting thresholds for appreciable benefit or harm remains challenging [42]–some might argue how relevant and translatable a clinical threshold for animal evidence is. Another challenge, analogous to that in human studies, is describing imprecision when a meta-analysis is not undertaken.

In the probiotics for pancreatitis example, the 95% CI around the Odds Ratio (OR) for mortality was wide, ranging from 0.24 to 1.22 and, applying GRADE’s guidance it included appreciable health benefit and harm possibly altering a clinical decision. The total sample size was small (102 animals in 3 studies). Thus, one would certainly rate down for imprecision.

Publication bias is a systematic underestimation or overestimation of the underlying beneficial or harmful effect due to the selective publication of studies. GRADE suggests considering downgrading for publication bias if the majority of studies was relatively small with positive results and commercially funded or if the review authors failed in conducting a comprehensive search.

Sena et al used the CAMARADES database to identify systematic reviews of animal studies of acute ischemic stroke and investigated the existence and impact of publication bias in these SRs. Based on Egger regression and trim-and-fill analysis the authors concluded that publication bias was likely and treatment efficacy was over estimated [43]. Korevaar and colleagues investigated how often systematic reviewers of laboratory animal experiments evaluate publication bias. Of the 35 included meta-analyses publication bias was considered in 74%, and in 60% the authors tried to formally assess it by funnel plot only or funnel plot and Egger’s test [44, 45]. Whether publication bias in preclinical animal studies can be assessed reliably similar to the clinical setting is open for discussion. The lack of formal registration of planned animal studies and generally small numbers of included studies pose challenges for the assessment of publication bias [46].

In the probiotics for pancreatitis example, we could not formally assess publication bias because of the small number of studies. It might be worthwhile to also consider the grey literature, contacting experts about unpublished studies and reviewing conference abstracts, which we did not do for this example.

#### Step 5b: Assess indirectness

Direct evidence comes from research that directly compares the interventions in which we are interested when applied to the populations in which we are interested and measures outcomes important to patients [22]. Certainty in the evidence may decrease when substantial differences exist between the population, the intervention, or the outcomes measured in relevant research studies and those under consideration in the clinical PICO in a SR.

In the context of GRADE for animal studies two layers of indirectness are proposed: the first layer considers indirectness from preclinical animal studies to the preclinical animal PICO. The second layer considers indirectness from animal models (preclinical animal studies) to humans (clinical PICO). This second layer of indirectness is called translatability (see Box 2).

An alternative approach is to consider all indirectness as one factor and to relate the evidence that was found directly to the clinical PICO, as the purpose of synthesizing the animal research was answering a clinical question.

S2 File provides a preliminary list of items related to indirectness. This list is largely based on the work by Henderson et al who identified threats to external/construct validity in preclinical research guidelines [47] and supplemented by our own findings.

An important challenge is to make a judgement about the choice of animal model. For example, does a ‘lower’ animal model representing the same metastasis-associated protein as humans (e.g. transgenic mouse) better reflect clinical pathophysiology than a ‘higher’ animal model expressing the species-specific metastasis-associated protein (e.g. pig)? Different animal models represent different aspects of the disease, and animal models that reflect all aspects of the clinical disease in one model are rare.

In the probiotics for pancreatitis example, there was serious indirectness. For the first layer of the two-layer approach, the timing of inducement of disease causes indirectness, as in some studies probiotics were administered before onset of the disease. The preclinical PICO aimed for probiotic treatment in animals with acute pancreatitis. In addition, one can debate if some of the animal experiments used a model for multi organ failure instead of acute pancreatitis.

For the second layer, indirectness of preclinical evidence to clinical PICO (translatability), histological damage and bacterial translocation were assessed as a measure for loss of function and infectious complications. However, these are surrogate outcomes for patient important outcomes, e.g., histological damage does not necessarily mean loss of function. In the clinical context, our question addresses probiotics administered to patients with severe acute pancreatitis, whereas in most animal studies the probiotics were administered before induction of pancreatitis. Acute pancreatitis was induced in various ways in the included animal models (e.g. sodium taurocholate intradermal, arginine intraperitoneal, glycodeoxycholate intradermal + cerulein intravenous, etc). Because there is no guidance available yet on how to decide which animal model reflects the clinical situation best, we did not take this into account in this example. This guidance will be developed and published in a future paper.

#### Step 5c: Assess upgrading

In the GRADE approach, non-randomized (observational) studies start as low certainty evidence, because of the high risk of confounding bias. In some situations however, upgrading the certainty from low to moderate (and perhaps even to high) certainty is appropriate.[23] The factors for upgrading include large magnitude of effect, presence of dose response relationship and opposing direction of plausible residual confounding.

Although most preclinical animal studies are experiments, upgrading may be relevant. The concept of upgrading might be different and warrants further research. For example, upgrading could be relevant where effects are consistent across different species. The US Food and Drug Administration (FDA) prefers and, in many cases requires testing of candidate drugs on at least two species (one rodent and one non-rodent) before moving on to human clinical trials [48]. In our expert meetings and in the environmental health field, consistency in the results between animal species and models is suggested as an upgrading factor [49]. A point of discussion is whether consistency across species should be seen as an upgrading factor, or as a component of inconsistency, indirectness/translatability.

#### Step 5d: Rating the certainty in the evidence

To arrive at a rating of the certainty in the evidence (high, moderate, low or very low), all eight factors need to be considered [50]. Similar to GRADE for human studies, the judgment on integrating the factors should be made explicit and transparent. A question could be, for example, how indirectness should be weighted in the overall rating of the evidence.

In the probiotics for pancreatitis example, we had serious concerns about risk of bias and imprecision for the outcome mortality. As a result, we downgraded the evidence by two levels, from high to low certainty in the evidence. We downgraded the evidence by an additional level, from low to very low, because of indirectness.

## Discussion

We presented a first version of the GRADE approach for assessing the certainty in the evidence from preclinical animal studies in the context of therapeutic interventions. In general, the generic GRADE approach appeared to be applicable. Our analysis of aspects of certainty in the evidence in a sample of systematic reviews and examples (methods, step C), however, showed that further operationalization of some of the GRADE domains is needed. For example, while rating down the certainty level due to risk of bias works similarly, the judgment of indirectness requires taking into account translatability to the clinical situation. We could not identify any additional downgrading factors. Consistency of effects across species was suggested as an additional upgrading factor.

One of the aims of this paper is to highlight methodological challenges. We identified several: 1) summarizing the findings of the animal evidence synthesis (approach to baseline risk and absolute effects, estimate of effect versus direction of effect), 2) calculating the OIS and defining clinical relevance thresholds (imprecision), 3) defining relevance of consistency within and across species (inconsistency), 4) specification and defining translatability/indirectness, and 5) the criteria for upgrading.

Regarding the probiotics example, we conclude for now that our certainty in the evidence for mortality was very low. In light of the methodological challenges, however, the final rating for certainty of the evidence might be different. Based on our certainty in the evidence assessment in this paper, proceeding to clinical trials would probably have been deemed premature. We would be inclined suggesting that more well-designed, executed and reported animal studies were necessary for a more valid estimation of the treatment effect in humans.

This conclusion is somewhat preliminary as no other factors were taken into account when going from evidence to decision. Developing an EtD framework for grading the strength of recommendations in the context of animal studies is a topic for further research. For example, in the decision to proceed to a first clinical trial for an intervention with therapeutic potential, evidence from animal studies provides only one of a number of evidence domains relevant to this decision. Other considerations include findings from in vitro research, human ex vivo studies, human genome wide association studies (which may confirm the importance of a pathophysiological target) and similarities (in terms of physiochemical structures or side effect profile) with drugs known to be effective. Each of these should be considered, with the same rigor, to inform optimal decision-making.

Although this is, as far as we are aware, the first paper on application of the GRADE approach to preclinical animal intervention studies, comparable approaches come from the field of environmental health [35]. The Navigation Guide and the US National Toxicology Program have developed frameworks for appraising animal toxicology data that include a GRADE-based assessment of the certainty in the evidence [49, 51]. The environmental health frameworks include animal studies as one of multiple evidence streams that are considered, followed by integration across the different evidence streams (e.g. also in vitro studies, observational studies and RCTs). The focus of environmental health questions is to identify health hazards and harms from environmental exposures, while our focus is on efficacy and safety of medical interventions in this case. Therefore, our framework will have similarities with methods developed for environmental health or toxicology.

GRADE provides a framework for a systematic, transparent and explicit assessment of the certainty in the evidence and strength of recommendations. Applying this approach to preclinical animal research could contribute to reducing research waste, either in animals or patients, and perhaps also to unraveling the mechanisms behind the low translational success rates of animal studies.

A potential barrier to implementation of the GRADE framework in animal studies is the lack of transparent reporting [52, 53]. The use of available reporting guidelines (such as ARRIVE [54, 55]) is generating more interest and should continue to be encouraged to further contribute to the optimal use of animal studies and better translation to clinical practice.

In summary, we tailored to the GRADE approach to assessing the certainty in the evidence from preclinical animal intervention studies. Further work will concentrate on performing case studies, addressing the identified methodological challenges and on creating a research agenda. We recently established a multidisciplinary GRADE for animal studies project group (as part of the GRADE working group) that has started to further develop the framework and work on case studies such as the example presented here. This group will discuss strategies and pitfalls with developing GRADE based frameworks in a variety of research fields (such as environmental health [49, 51, 56–58], toxicology and medicine) for improving application of the GRADE approach to preclinical animal intervention studies. Although further methodological work is needed, the generic GRADE approach appeared applicable and the presented preclinical animal intervention studies GRADE framework provides the much-needed guidance to improve interpreting of the results of SRs of animal studies and to rate the certainty in the evidence. This framework will help not only researchers but also potential funders of clinical or animal research and ethical committees, thereby reducing the risk of misinforming future human studies.

## Supporting information

S1 Table Summary of identified criteria possibly related to certainty in the evidence resulting from analysis of the paper of Krauth et al. (XLSX)

S2 Table Summary of identified criteria possibly related to certainty in the evidence in the methods and discussion sections of SRs of preclinical animal studies. (XLSX)

S3 Table Summary of all (unduplicated) criteria possibly related to certainty in the evidence. (XLSX)

S4 Table Main differences between risk of bias of clinical and preclinical animal intervention studies. (DOCX)

S1 File Attendance at the expert meetings. (DOCX)

S2 File Examples of assessing indirectness of evidence from preclinical animal studies. (DOCX)
