# Supplementary material for: Facilitating healthcare decisions by assessing the certainty in the evidence from preclinical animal studies
Source: PLoS One. 2018 Jan 11;13(1):e0187271. doi: 10.1371/journal.pone.0187271 (PMC5764235; doi:10.1371/journal.pone.0187271)
Supplement: S4 Table — (DOCX) [file pone.0187271.s004.docx]

**S4 Table: Main differences between risk of bias of clinical and preclinical animal intervention studies**

| **Type of bias** | **Domain** | **Description in Cochrane Collaboration's RoB tool** | **Description in SYRCLEs RoB tool** | **Argumentation for difference between tools** |
| --- | --- | --- | --- | --- |
| Selection bias | Random sequence generation | Selection bias (biased allocation to interventions) due to inadequate generation of a randomised sequence. | Was the allocation sequence adequately generated and applied? | Comparable |
| Selection bias | Similarity at baseline |  | Were the groups similar at baseline or were they adjusted for confounders in the analysis? | New: Random allocation of animals to the experimental and control groups, is not yet standard practice in animal experiments. Furthermore, as the sample size of most animal experiments is relatively small, mportant baseline differences may be present. Therefore, the assessment of similarity in baseline characteristics between the experimental and control groups are proposed to be a standard item. |
| Selection bias | Allocation concealment | Selection bias (biased allocation to interventions) due to inadequate concealment of allocations prior to assignment. | Was the allocation adequately concealed? | Comparable |
| Performance bias | Random housing |  | Were the animals randomly housed during the experiment? | New: In animal studies, the investigators are responsible for the way the animals are housed. They determine, for example, the location of the cage in the room. As housing conditions (such as lighting, humidity, temperature, tc.) are known to influence study outcomes (such as certain biochemical parameters and behavior), it is important that the housing of these animals is randomized or, in other words, comparable between the experimental groups in order to reduce bias. |
| Performance bias | Blinding of participants and personnel | Performance bias due to knowledge of the allocated interventions by participants and personnel during the study. | Were the caregivers and /or investigators blinded from knowledge which intervention each animal received during the experiment? | Comparable |
| Detection bias | Random outcome assessment |  | Were animals selected at random for outcome assessment? | New: A reason to select animals at random for outcome assessment is the presence of circadian rhythms in many biological processes. Not selecting the animals for outcome assessment at random might influence the direction and magnitude of the effect. Random outcome assessment is also important to assure blinding of the outcome assessment |
| Detection bias | Blinding of outcome assessment | Detection bias due to knowledge of the allocated interventions by outcome assessors. | Was the outcome assessor blinded? | Comparable |
| Attrition bias | Incomplete outcome data | Attrition bias due to amount, nature or handling of incomplete outcome data. | Were incomplete outcome data adequately addressed? | Comparable |
| Reporting bias | Selective reporting | Reporting bias due to selective outcome reporting. | Are reports of the study free of selective outcome reporting? | Comparable |
| Other bias | Other bias | Bias due to problems not covered elsewhere in the table. | Was the study apparently free of other problems that could result in high risk of bias? | Comparable |
