# Supplementary material for: Facilitating healthcare decisions by assessing the certainty in the evidence from preclinical animal studies
Source: PLoS One. 2018 Jan 11;13(1):e0187271. doi: 10.1371/journal.pone.0187271 (PMC5764235; doi:10.1371/journal.pone.0187271)
Supplement: S1 File — (DOCX) [file pone.0187271.s005.docx]

**S1 File: Attendance at the expert meetings**

**Expert meeting 1:**

Maroeska Rovers, Merel Ritskes-Hoitinga, Mariska Leeflang, Lotty Hooft, Joanna in ‘t Hout, Rob de Vries, Miranda Langendam, Carlijn Hooijmans

**Expert meeting 2:**

Maroeska Rovers, Merel Ritskes-Hoitinga, Mariska Leeflang, Joanna in ‘t Hout, Rob de Vries, Miranda Langendam, Carlijn Hooijmans, Hans de Beer

**Individual expert meetings:**

Holger Schünemann (multiple meetings, in person and Skype)

Joanna in ‘t Hout

Maroeska Rovers

Mariska Leeflang

Malcolm Macleod, Emily Sena, Gillian Currie

Kristina Thayer (Skype)

Andrew Rooney (Skype)
