# Supplementary material for: Facilitating healthcare decisions by assessing the certainty in the evidence from preclinical animal studies
Source: PLoS One. 2018 Jan 11;13(1):e0187271. doi: 10.1371/journal.pone.0187271 (PMC5764235; doi:10.1371/journal.pone.0187271)
Supplement: S2 File — (DOCX) [file pone.0187271.s006.docx]

**S2 File: Examples of assessing indirectness of evidence from preclinical animal studies**

**The population or the induced disease in the studies differs substantially from the population or the disease in which we are interested**

- Matching preclinical conditions to clinical setting (also with regard to physiological derangement; i.e. should ideally be similar)
- Assessment of multiple manifestations of disease phenotype
- Species: multiple species tested; comparable results between different species
- Animal model/disease: similarity of disease in clinical setting, e.g. how is disease induced in the animals
- Model match to human manifestation of disease, interventions and sex, age and co-morbidities of patients in clinical setting
- Characterization of animal properties at baseline
- Co-morbidities

**The Intervention of the studies differs substantially from intervention in which we are interested**

- Optimization of complex treatment parameters
- Matching timing of treatment delivery to clinical setting
- Matching route/method of treatment delivery to clinical setting
- Definition of treatment
- Faithful delivery of intended treatment
- Theoretical relationship between experimental operations/interventions and clinical scenario
- Treatment response along mechanistic pathway
- Use of validated assay for molecular pathways assessment
- Treatment interactions with clinically relevant co-morbidities

**The Comparison of the studies differs substantially from comparison in which we are interested**

- Appropriate control group
- Comparability of control group characteristics to those of previous studies
- Indirect comparisons

**The Outcome of the studies differs substantially from outcome in which we are interested**

- Degree of characterization and validity of outcome measure chosen
- Assessment of outcome at late/clinically relevant time points
